# Sanguinarine Inhibits the 2-Ketogluconate Pathway of Glucose Utilization in *Pseudomonas aeruginosa*

**DOI:** 10.3389/fmicb.2021.744458

**Published:** 2021-09-10

**Authors:** Federica A. Falchi, Giorgia Borlotti, Francesco Ferretti, Gianvito Pellegrino, Matteo Raneri, Marco Schiavoni, Alessandro Caselli, Federica Briani

**Affiliations:** ^1^Dipartimento di Bioscienze, Università degli Studi di Milano, Milan, Italy; ^2^Dipartimento di Chimica, Università degli Studi di Milano, Milan, Italy

**Keywords:** bacterial infections in hyperglycemic patients, *Pseudomonas aeruginosa*, glucose catabolism, antibacterial drug discovery, drug repurposing, Prestwick Chemical Library, sanguinarine

## Abstract

Interfering with the ability of pathogenic bacteria to import glucose may represent a new promising antibacterial strategy, especially for the treatment of infections occurring in diabetic and other hyperglycemic patients. Such patients are particularly susceptible to infections caused by a variety of bacteria, among which opportunistic pathogens like *Pseudomonas aeruginosa*. In *P. aeruginosa*, glucose can be directly imported into the cytoplasm or after its periplasmic oxidation into gluconate and 2-ketogluconate (2-KG). We recently demonstrated that a *P. aeruginosa* mutant lacking the 2-KG transporter KguT is less virulent than its *kguT*^+^ parental strain in an insect infection model, pointing to 2-KG branch of glucose utilization as a possible target for anti-*Pseudomonas* drugs. In this work, we devised an experimental protocol to find specific inhibitors of the 2-KG pathway of *P. aeruginosa* glucose utilization and applied it to the screening of the Prestwick Chemical Library. By exploiting mutants lacking genes involved in the transport of glucose derivatives in the primary screening and in the secondary assays, we could identify sanguinarine as an inhibitor of 2-KG utilization. We also demonstrated that sanguinarine does not prevent 2-KG formation by gluconate oxidation or its transport, suggesting that either KguD or KguK is the target of sanguinarine in *P. Aeruginosa*.

## Introduction

*Pseudomonas aeruginosa* is a common environmental Gram-negative bacterium that behaves as an opportunistic pathogen in humans. It typically infects the pulmonary and urinary tracts, burns, and wounds; and almost all clinical cases of *P. aeruginosa* infections occur in compromised hosts (Lyczak et al., 2000). Due to the low permeability of its outer membrane, *P. aeruginosa* is intrinsically resistant to different antibiotics. Such intrinsic resistance is increased by mutations and adaptive responses to antibiotic exposure, leading to the selection and diffusion of multidrug-resistant (MDR) strains that are very difficult to eradicate (Breidenstein et al., 2011).

In the last years, emphasis has been put on virulence genes (i.e., encoding functions specifically involved in the infection of the host, like host tissue adhesion or escape from immunity system) as new targets for antimicrobials (Karow et al., 1991). On the other hand, metabolic genes have been repeatedly identified in *in vivo* screenings for genes actually contributing to the virulence of relevant pathogens intrinsically resistant to antibiotics like *P. aeruginosa* (Handfield et al., 2000; de Lorenzo, 2015; Dubern et al., 2015). These findings can be rationalized by considering that for *P. aeruginosa*, and conceivably also for other pathogens, metabolic functions may deeply impact the infection process by determining adaptations to the specific nutritional environment of the host (de Lorenzo, 2015; Okon et al., 2017). This may explain (or at least contribute to) the wide variability in the virulence degree exhibited by *P. aeruginosa* isolates upon infection of diverse model hosts (Dubern et al., 2015; Hilker et al., 2015). Thus, metabolic functions appear as interesting and still largely overlooked targets for novel antibacterial strategies.

Pathologies leading to increased glucose concentration in plasma result in the augmented risk of developing serious infections by *P. aeruginosa* and other bacteria (Rayfield et al., 1982; Bodey et al., 1983; Muller et al., 2005; Garnett et al., 2013; Hobson et al., 2014). For instance, in diabetic patients, a poor control of glycemia is associated with an increased susceptibility to infections (Baker et al., 2006; Peleg et al., 2007; Burekovic et al., 2014). Hyperglycemia impairs the organism response to bacterial infections through different mechanisms, among which growth stimulation of bacteria that can utilize glucose as carbon and energy source. In agreement with this view, *P. aeruginosa* mutants defective in glucose metabolism were demonstrated to be less virulent in a hyperglycemic mouse model of acute lung infection (Gill et al., 2016). Moreover, unidentified mutations causing upregulated expression of the *zwf* gene coding for glucose-6-phosphate dehydrogenase, a key enzyme of glucose catabolism, have been reported (Silo-Suh et al., 2005) in *P. aeruginosa* clinical isolates from patients with cystic fibrosis (CF), who very frequently develop chronic *P. aeruginosa* lung infections.

Drugs interfering with the ability of pathogenic bacteria to import or catabolize glucose may thus represent promising co-adjuvants in antibiotic therapies of bacterial infections, with particular regard to those occurring in hyperglycemic patients. Given the high prevalence of diabetes in the elderly population, and since transient hyperglycemia develops also as a consequence of surgery or serious illness in non-diabetic patients (Xiu et al., 2014; Ceriello et al., 2020), finding inhibitors of glucose utilization by pathogenic bacteria may have broad implications.

**FIGURE 1:**
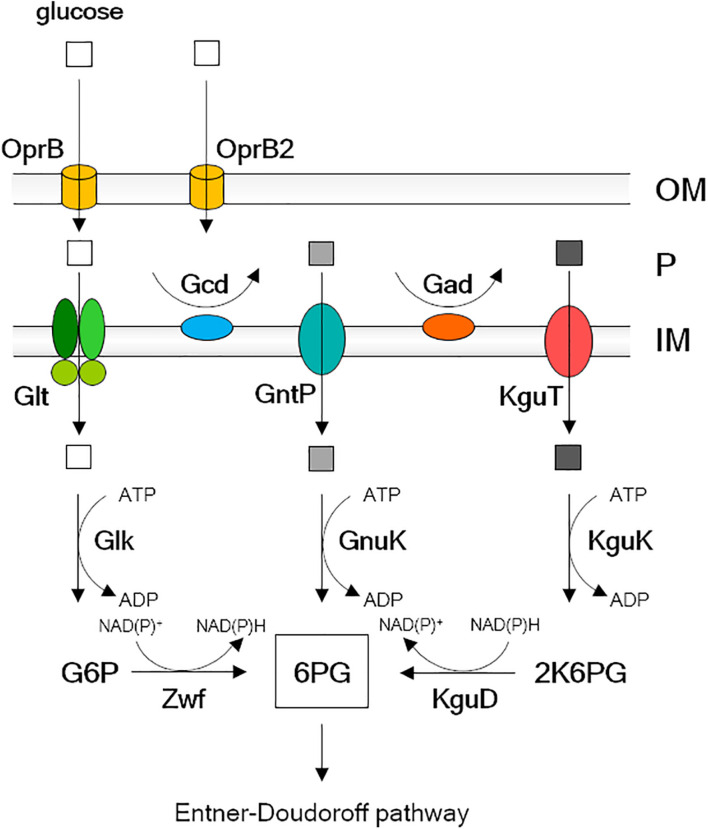
Glucose uptake pathway and transformation into 6-phosphogluconate in *Pseudomonas aeruginosa*. Explanation is in the Introduction text. OM, outer membrane; P, periplasm; IM, inner membrane; G6P, glucose-6-phosphate; 6PG, 6-phosphogluconate; 2K6PG, 2-keto-6 phosphogluconate. Empty square, glucose; light gray square, gluconate; dark gray square, 2-ketogluconate (2-KG).

*P. aeruginosa* can use glucose as the sole carbon source, although it is not its favorite one (Ng and Dawes, 1973; Whiting et al., 1976; Lessie and Phibbs, 2003; Valentini et al., 2014). Glucose crosses *P. aeruginosa* outer membrane through the OprB and OprB2 selective porins (Chevalier et al., 2017). Once in the periplasm, glucose can either be transported to the cytoplasm through the inner membrane by the ABC transporter GltF-GltG-GltK or be oxidized to gluconate by Gcd (Midgley and Dawes, 1973; Adewoye and Worobec, 2000). Gluconate can in turn enter the cytoplasm through the GntP transporter or be oxidized to 2-ketogluconate (2-KG), which enters the cytoplasm through the KguT transporter (Swanson et al., 2000; Figure 1). In the cytoplasm, the three import pathways converge on the synthesis of 6-phosphogluconate, which is metabolized through the Entner–Doudoroff pathway (Lessie and Phibbs, 2003).

In a previous work, we generated a collection of glucose uptake defective (GUD) mutants with single and multiple deletions of genes encoding Glt, GntP, and KguT (Raneri et al., 2018). Some of these mutants rely on a single glucose import route and can be thus useful in the research of specific inhibitors of glucose catabolism branches. The goal of this work was the identification of specific inhibitors of the 2-KG transport/utilization branch by applying a drug repurposing strategy. Our results indicate that sanguinarine, a benzophenanthridine alkaloid extracted from *Sanguinaria canadensis*, inhibits *P. aerugino*sa 2-KG utilization, most likely by interfering with the function of KguD or KguK proteins.

## Materials and Methods

### Bacterial Strains and Plasmids

Strains and plasmids used in this work are listed in Table 1. Bacterial cultures were grown in LD broth (10 g/L of tryptone, 5 g/L of yeast extract, and 5 g/L of NaCl) or M9tx minimal medium (0.1% NH_4_Cl, 1.6% Na_2_HPO_4_⋅12H_2_O, 0.3% KH_2_PO_4_, 0.5% NaCl, 0.013% MgSO_4_, 0.001% CaCl_2_, trace elements, and 0.05% Triton X-100). Solid media were prepared with LD10 (LD broth supplemented with 1% agar). When needed, media were supplemented with 0.4% (w/v) glucose, 0.4% (w/v) gluconate, ca. 0.2% (w/v) 2-KG, and 20 mM of succinate. 2-KG was prepared from calcium 2-KG as described (Swanson et al., 2000).

**TABLE 1:** *Pseudomonas aeruginosa* strains and plasmids.

**Laboratory strains**				
**Strain**	**Genotype**			**References**
PAO1	Reference strain			Stover et al., 2000
PAMO107	PAO1 Δ*gntP*			Raneri et al., 2018
PAMO108	PAO1 Δ*kguT*			Raneri et al., 2018

**Environmental strain**	**Origin**			**References**

E1	Salad			Bragonzi et al., 2009
E2	Salad			Bragonzi et al., 2009
E4	Salad			Bragonzi et al., 2009
E5	Red pepper			Bragonzi et al., 2009
E9	Crème			Bragonzi et al., 2009

**Clinical isolates**	**Patient**	**Disease^a^**	***P. aeruginosa* status**	**References**

AA2	AA	CF	Early infection	Bragonzi et al., 2009
AA43	AA	CF	Chronic, mucoid	Bragonzi et al., 2009
AA44	AA	CF	Chronic	Bragonzi et al., 2009
TR1	TR	CF	Early infection	Bragonzi et al., 2009
TR66	TR	CF	Chronic	Bragonzi et al., 2009
TR67	TR	CF	Chronic, mucoid	Bragonzi et al., 2009
CL1	CL	CF	Intermittent	Forti et al., 2018
CL2	CL	CF	Intermittent, mucoid	Forti et al., 2018
GS3	GS	CF	Intermittent, mucoid	Forti et al., 2018
DV4	DV	COPD	Chronic	Forti et al., 2018
GA7	GA	CF	Chronic, mucoid	Forti et al., 2018
VR8	VR	CF	Chronic	Forti et al., 2018
AA10	AA	COPD	Chronic	Forti et al., 2018
AG5	AG	CF	Chronic	Forti et al., 2018
GJY9	GJY	CF	Chronic	Forti et al., 2018

*^a^CF, cystic fibrosis; COPD, chronic obstructive pulmonary disease.*

### Prestwick Chemical Library Screening and Secondary Assay

The Prestwick Chemical Library (Prestwick Chemical Inc., Illkirch, France) was a kind gift of P. Seneci and P. Landini. The 1,120 library compounds are at 2 mg/ml concentration in 100% dimethyl sulfoxide (DMSO). For the first screening of the Prestwick Chemical Library and the secondary assays, PAMO107 (i.e., PAO1 Δ*gntP*) and PAO1 cultures in M9tx supplemented with either 0.4% glucose or gluconate were diluted to OD_600_ 0.01 in the same medium. Culture aliquots (145 μl) were distributed in the wells of 96-well plates, and 5 μl of Prestwick Chemical compounds was added (final concentration, 66.7 μg/ml). As controls, some microcultures were supplemented with 5 μl of DMSO or 33.0 μg/ml of ciprofloxacin. The plates were incubated at 37°C with slow agitation for 24 h, and the growth was monitored by measuring the OD_600_ at the EnSight microplate reader (PerkinElmer, Waltham, MA, United States). R was calculated as (A–B)/A, where A and B are the OD_600_ reached after incubation of the culture without (with DMSO only) and with sanguinarine, respectively.

### Sanguinarine Dose–Response Assay

Sanguinarine chloride (MW 367.78 g/mol; hereafter sanguinarine) stock solution was prepared at 2 mg/ml (i.e., 5.4 mM) in 100% DMSO and twofold serially diluted in DMSO to 0.0625 mg/ml. Overnight cultures of PAMO107 in LD were washed twice in M9tx with 0.4% gluconate and resuspended in 1 ml of the same medium at OD_600_ 0.01 in glass test tubes. Cultures were incubated 24 h at 37°C in the presence of 33 μl of either 2 mg/ml or diluted sanguinarine samples (or the same volume of DMSO for control cultures). The OD_600_ was measured at the EnSight reader (PerkinElmer). The 50 and 90% inhibitory concentration (IC_50_ and IC_90_) were estimated as the sanguinarine concentrations that reduced the OD_600_ by 50 and 90%, respectively, compared with that of the control culture with DMSO. Three biological replicates were analyzed for each strain/condition.

### Microscopy for Bacterial Cells Observation

PAO1 cultures grown in M9tx supplemented with 0.4% gluconate and either 66.6 μg/ml of sanguinarine chloride in DMSO or the same volume of DMSO only were fixed with formaldehyde (0.37% final concentration) for 30 min at 37°C with shaking. Cells were pelleted, washed with phosphate-buffered saline (PBS) buffer, and resuspended in 1/10 volume of PBS. Cell suspension (10 μl) was layered on microscope slides precoated with a thin layer of 1.5% agarose and observed with a Leica DMRA2 widefield microscope (Leica Microsystems, Wetzlar, Germany) using the 100 × immersion objective. Fluorescence of sanguinarine was detected in the UV channel (excitation–emission filters, 360/40–470/40).

### Sanguinarine Quantification in Spent Medium

Samples (5 ml) of M9tx medium supplemented with 0.4% gluconate and 66.6 μg/ml of sanguinarine in glass tubes were mock-inoculated or inoculated with PAO1, PAMO107, or PAMO108 at OD_600_ = 0.01 and incubated overnight at 37°C with gentle agitation. The cells in the supernatants were removed by centrifugation, and the supernatants were filtered through 0.45-μm pore size filters. Supernatants (4 ml) were freeze dried. The solid residue was extracted with ca. 1 ml of MeOH [high-performance liquid chromatography (HPLC) grade] by stirring in a sealed flask for 1 h. MeOH was added by micropipette; but for reliability and precision of sanguinarine quantification, the MeOH amount was measured by weight. The suspension was then filtered on a 0.45-μm syringe filter, diluted up to 10 ml, and analyzed by HPLC in triplicate. The analyses were performed using a Kinetex^®^ -5μm C18 100 Å column (150 mm × 4.6 mm, i.d.) on a JASCO PU4180 equipped with a MD4015 PDA detector. A fixed 1 ml/min flow rate of the mobile phase was used. The analyses were performed with gradient elution using a mobile phase consisting of a solvent A (0.1% formic acid in deionized water) and a solvent B (0.1% formic acid in acetonitrile). The following program was employed: 20% B at 0–5 min, 21–90% B at 5–19 min, maintained 90% B for 10 min, and then 90–20% B in 3 min. The injection volume was 20 μl. Sanguinarine signal (retention time 9.55 min) was integrated at the wavelength of 254 nm. For the calibration of sanguinarine, standard solution in MeOH (HPLC grade) was initially prepared; however, the resulting calibration curve was not reliable due to a marked matrix effect of the residue of the medium extracted during sample preparation. Thus, a calibration was performed using the cell culture medium (Supplementary Figure 1). One milliliter of a stock solution of sanguinarine in DMSO (6.7 mM) was added to 18.5 ml of the medium and stirred thoroughly. The volatiles were then evaporated under vacuum, and the solid residue extracted three times with 2 ml of MeOH. The combined extractions were collected in a 10-ml measuring flask and made up to the mark. Four standard calibration solutions of sanguinarine ranging from 98.2 to 12.3 μg/ml were prepared by dilution of the mother solution and injected in triplicate.

### Ketogluconate Uptake Assay

2-KG was measured in cell-free supernatants as described (Lanning and Cohen, 1951). In brief, overnight cultures of PAO1 and PAMO108 were centrifuged, and the bacterial cells washed twice with M9tx and diluted to OD_600_ = 0.01 in M9tx supplemented with 0.2% 2-KG and 0.4% gluconate. Cultures were grown to OD_600_ = 0.2–0.3 at 37°C, and 1.0 OD_600_ (corresponding to ca. 2 × 10^8^ cfu) samples were withdrawn and centrifuged. Bacterial pellets were washed twice in M9tx, resuspended in 1 ml of M9tx containing 15 μg/ml of 2-KG, and supplemented with either 33 μl of 2 mg/ml of sanguinarine in DMSO (final concentration 66.6 μg/ml) or an equal volume of DMSO and incubated 30 min at 37°C with shaking. The samples were centrifuged 5 min at 12,000 × g, and the cell-free supernatants were mixed with 0.5 ml of a freshly prepared 15 mg/ml solution of *o*-phenylenediamine in 0.25 N of HCl. The reaction mixtures were heated 30 min at 100°C in a boiling water bath and cooled to room temperature. Absorbance at 330 nm (A_330_) was read in a spectrophotometer and used to determine the 2-KG concentration by comparison with standard curves generated by testing samples containing known 2-KG concentrations in M9tx and DMSO as described above. Since sanguinarine absorbs light at 330 nm (Janovská et al., 2009), the A_330_ of samples without 2-KG and with sanguinarine was considered as background absorbance and subtracted from the A_330_ of all sanguinarine-containing samples (see Supplementary Figure 2 for the assay description and 2-KG standard curves obtained in the presence or absence of sanguinarine).

## Results

### Searching for 2-Ketogluconate Metabolism Inhibitors: Rationale

*P. aeruginosa* proteins essential for growth with 2-KG as the sole carbon source, but dispensable for growth on gluconate or glucose, are the KguT transporter and the KguK and KguD cytoplasmic enzymes. In addition, a Δ*gntP* mutant strain growing with gluconate as the sole carbon source would require also Gad, which oxidizes gluconate to 2-KG in the periplasm, besides the three above proteins (Figure 1). Thus, the growth of a Δ*gntP* mutant on minimal medium with gluconate as carbon source would be prevented by molecules inhibiting one out of these four proteins. On the contrary, such molecules should not interfere with the Δ*gntP* mutant growth on glucose, which enters the cell due to Glt and is converted into 6-phosphogluconate by Glk and Zwf (Figure 1). Thus, through a simple two-step screening based on the differential growth of a Δ*gntP* strain in media containing either glucose or gluconate as carbon source, it would be possible to identify specific inhibitors of the 2-KG branch of glucose utilization.

### Searching for 2-Ketogluconate Metabolism Inhibitors: Primary Screening

In the primary screening, the 1,120 compounds of the Prestwick Chemical Library were tested for their ability to inhibit the growth of the PAO1 Δ*gntP* mutant in minimal medium with gluconate as the sole carbon source. The growth was performed in 96-well plates and was evaluated by measuring the OD_600_ before (OD_60__0_^t0^) and after (OD_60__0_^t24^) 24 h of incubation at 37°C in the presence of the Prestwick compounds. One hundred fourteen compounds that did interfere with growth (i.e., with OD_60__0_^t24^ - OD_60__0_^t0^ ≤ 0.08) were found in the primary screening. Among them, 25 had already known antibacterial activity (i.e., antibiotics, antibacterial, anti-infective, and antiseptic compounds) and were not further analyzed. The 89 remaining compounds (Supplementary Table 1) were subjected to further analyses.

### Searching for 2-Ketogluconate Utilization Inhibitors: Secondary Screening

Among the compounds selected in the primary screening, inhibitors of different metabolic routes and processes essential to sustain growth in minimal medium should be present. In order to find specific inhibitors of gluconate oxidation and/or 2-KG import, we applied a secondary screening; and in particular, we tested (i) the growth of Δ*gntP* strain with gluconate (i.e., in the same conditions of the primary screening) to confirm the results of the primary screening and select compounds with the highest inhibitory activity. We considered as inhibitors the compounds determining a ≥ 80% growth reduction with respect to the growth of the control culture with DMSO only; and (ii) the growth of the Δ*gntP* strain with glucose and of the *gntP*^+^ PAO1 strain with gluconate, which in both cases does not require Gad, KguT, KguD, or KguK.

**FIGURE 2:**
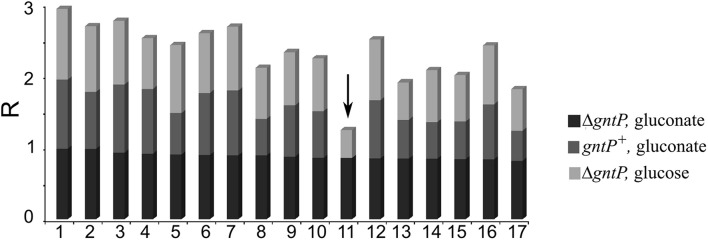
Secondary screening on putative 2-ketogluconate (2-KG) utilization inhibitors. PAO1 (*gntP*^+^) or PAMO107 (Δ*gntP*) cultures (150 μl) were grown 24 h at 37°C in 96-well plates in M9 supplemented with 0.4% glucose or gluconate, as indicated on the right, and 5 μl of the following compounds at a final 66.6 μg/ml concentration: 1, ciclopirox ethanolamine; 2, flucytosine; 3, clioquinol; 4, azacyclonol; 5, 8-azaguanine; 6, cyproheptadine hydrochloride; 7, paroxetine hydrochloride; 8, atovaquone; 9, bepridil hydrochloride; 10, acacetin; 11, sanguinarine (arrow); 12, promethazine hydrochloride; 13, fluvoxamine maleate; 14, metergoline; 15, amethopterin (*R*,*S*); 16, promazine hydrochloride; 17, methiothepin maleate. Growth was estimated by reading the OD_600_. R was calculated for each strain/condition with respect to control cultures containing 5 μl of DMSO as explained in section “Materials and Methods”. *R* = 1, complete growth inhibition; *R* = 0, no inhibition.

Seventeen compounds were found to inhibit the growth of the Δ*gntP* strain with gluconate by at least the 80% (Figure 2). The compound showing the best performance (i.e., preventing the Δ*gntP* strain growth with gluconate and affecting to a limited extent the growth of the Δ*gntP* strain with glucose or of the wt PAO1 strain with gluconate) was sanguinarine (Figure 2, arrow), which was further analyzed.

We also observed that 30 compounds reduced the growth of the Δ*gntP* strain with both glucose and gluconate and of the *gntP*^+^ PAO1 strain with gluconate by at least 50% (Supplementary Table 2), showing thus growth inhibition in minimal medium irrespective of the carbon source.

### Evaluation of Sanguinarine Dose–Response Effect and Specificity

**FIGURE 3:**
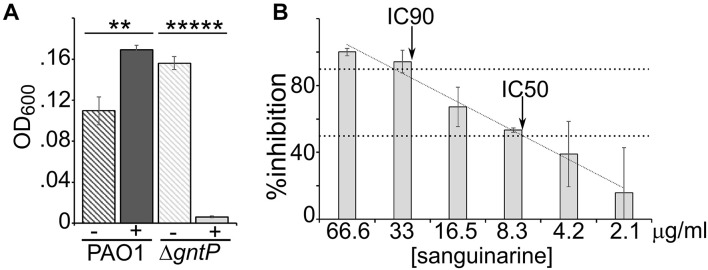
Dose–effect of sanguinarine on growth. **(A)** Growth of PAO1 or PAMO107 (PAO1 Δ*gntP*) at 37°C in 1 ml of M9tx with 0.4% gluconate in absence (−) or presence of 66.6 μg/ml of sanguinarine. Bars represent average with standard deviation (*N* = 3). Significance of the difference between growth with and without sanguinarine was evaluated with *t*-test. **p < 0.01; *****p < 0.00001. **(B)** Growth of PAMO107 at 37°C in M9tx with 0.4% gluconate with the sanguinarine concentrations indicated below the histogram. In both panels, bars represent average with standard deviation (*N* = 3). **(B)**% Inhibition was calculated as R × 100. The arrows indicate the points where the inhibitory concentration trendline (*R*^2^ = 0.98) crosses the two horizontal dashed lines corresponding to 50% (IC_50_) and 90% (IC_90_) inhibition.

The effect of sanguinarine on the growth of PAO1 and its Δ*gntP* derivative was tested in aerated cultures growing in minimal medium with gluconate as the carbon source. We confirmed that also in such growth conditions (i.e., in 1 ml cultures in glass tubes instead of 96-well plates), sanguinarine inhibited the growth of the Δ*gntP* mutant, whereas it did not prevent the growth of PAO1, which was slightly stimulated (Figure 3A). With respect to the Δ*gntP* mutant growing in gluconate medium, we found that sanguinarine had IC_50_ between 8.3 and 4.2 μg/ml (i.e., between ca. 22 and 11 μM), whereas the IC_90_ was between 66.6 and 33.0 μg/ml (Figure 3B).

### Sanguinarine Does Not Target Gad

**FIGURE 4:**
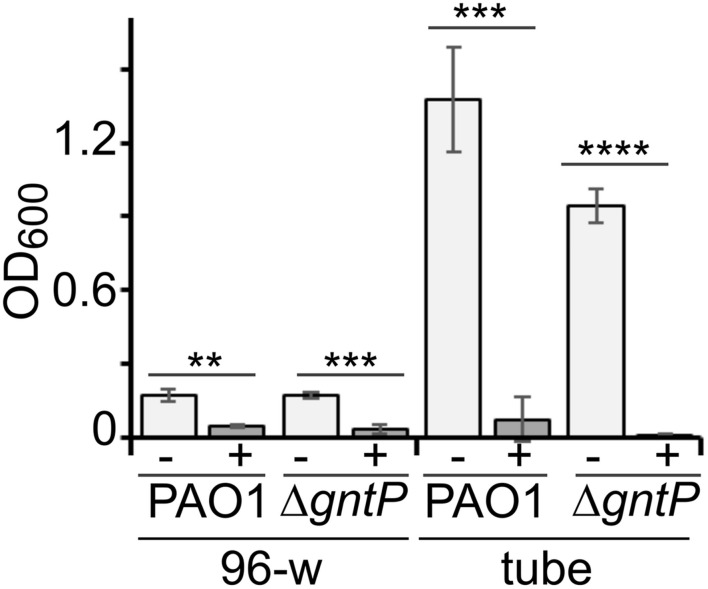
Effect of sanguinarine on 2-ketogluconate (2-KG) utilization. Growth of PAO1 or PAMO107 (PAO1 Δ*gntP*) in M9tx with 0.2% 2-KG in absence (−) or presence of sanguinarine at 66.6 μg/ml (+) at 37°C in 96-well plates (96-w; incubation = 48 h) or glass tubes (tube; incubation = 24 h). Bars represent average with standard deviation (*N* = 3). Significance of the difference between growth with and without sanguinarine was evaluated with *t*-test. **p < 0.01; ***p < 0.001; ****p < 0.0001.

Sanguinarine may prevent growth on gluconate of the Δ*gntP* strain by inhibiting either factors involved in 2-KG import and utilization (namely, KguT, KguD, or KguK) or the Gad protein, which converts gluconate into 2-KG (Figure 1). In the first case (i.e., inhibition of KguT, KguD, or KguK), both PAO1 and the Δ*gntP* strain would not grow in the presence of 2-KG as the carbon source and sanguinarine (Raneri et al., 2018). On the other hand, if Gad were the putative sanguinarine target, both strains would grow with 2-KG as the carbon source irrespective of the presence of sanguinarine. As shown in Figure 4, we found that sanguinarine inhibits the growth of both PAO1 and its Δ*gntP* derivative in the presence of 2-KG as the sole carbon source. Thus, sanguinarine does not inhibit gluconate oxidation by Gad, but either 2-KG transport through KguT or 2-KG conversion into 6-phosphogluconate by KguD/KguK.

### Sanguinarine Does Not Inhibit 2-Ketogluconate Transport

**FIGURE 5:**
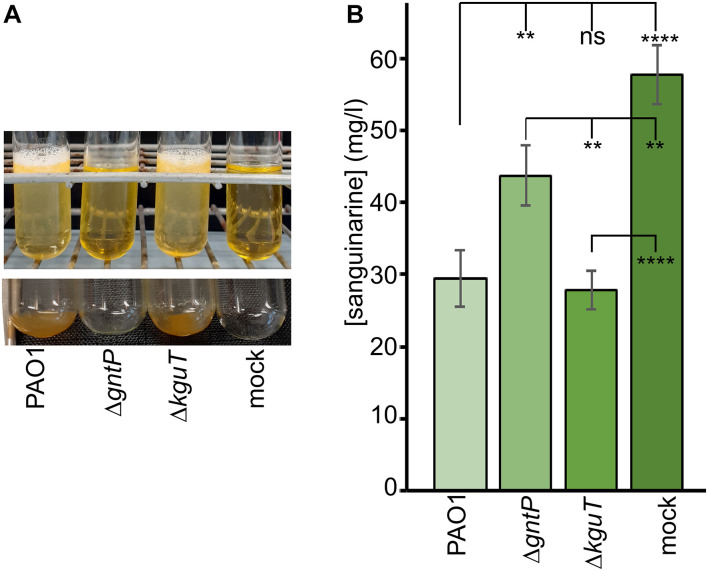
Sanguinarine concentrations in spent medium. The spent medium of PAO1, PAMO107 (Δ*gntP*), and PAMO108 (Δ*kguT*) cultures incubated overnight at 37°C in M9tx with 0.4% gluconate and 66.6 μg/ml of sanguinarine was analyzed by high-performance liquid chromatography (HPLC) as described in section “Materials and Methods”. As a control, sterile medium incubated as described above was also analyzed (mock). **(A)** Cultures as they appeared after the incubation (upper panel) and the tubes after cell resuspension by pipetting and removal of the supernatant (lower panel). **(B)** HPLC quantification of sanguinarine in the spent medium. Results are the average of three determinations on independent cultures with standard deviation. Stars refer to P estimated by ANOVA and Tukey’s *post hoc* test. **p < 0.01; ****p < 0.0001; ns, not significant.

To check whether sanguinarine could enter *Pseudomonas* cells, we measured by HPLC its concentration in the spent medium of PAO1, Δ*gntP*, and Δ*kguT* cultures grown in M9tx supplemented with gluconate and sanguinarine. As expected, in the growth conditions of the assay (5 ml of cultures in glass tubes), the Δ*gntP* mutant could not grow. Instead, the other two strains formed a biofilm stuck to the tube bottom, which could not be detached by vortexing the tube (Figure 5A). We found that sanguinarine concentration in the spent medium of PAO1 and Δ*kguT* was halved with respect to that measured in the mock (i.e., without bacterial cells) samples. A 25% decrease was observed in the medium of Δ*gntP* cultures, in spite of very poor growth that reduced the number of cells potentially able to import sanguinarine in the cultures of this mutant with respect to PAO1 and Δ*kguT* ones (Figures 5A,B). These results suggest that sanguinarine may enter *P. aeruginosa* cells. In agreement with this hypothesis, PAO1 cells grown in the presence of sanguinarine are homogenously fluorescent (Supplementary Figure 3), a phenotype reasonably due to the entry in the cytoplasm of sanguinarine, which, like other alkaloids, is fluorescent (Janovská et al., 2009).

**FIGURE 6:**
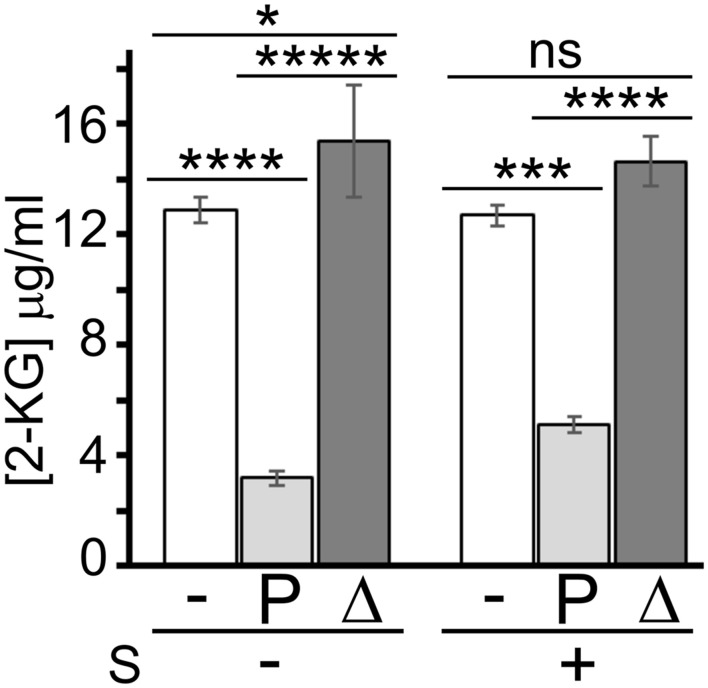
Effect of sanguinarine on 2-ketogluconate (2-KG) uptake. Concentration of 2-KG remaining in the medium after 30-min incubation with PAO1 (P) and PAO1 Δ*kguT* (Δ) or without bacteria (−) in the presence (+) or absence (−) of 66.6 μg/ml of sanguinarine (S). 2-KG was added to the medium at 15 μg/ml. Bars represent average of the results obtained on three independent cultures with standard deviation. Stars refer to P estimated by ANOVA and Tukey’s *post hoc* test. *p < 0.05; ***p < 0.001; ****p < 0.0001; *****p < 0.00001; ns, not significant. The difference between PAO1 samples with or without sanguinarine was significant according to *t*-test (p < 0.001).

To test whether sanguinarine may inhibit the KguT-dependent 2-KG transport, preventing in this way the growth on this carbon source, we performed a 2-KG uptake assay. We measured 2-KG concentration in the growth medium with a colorimetric assay (Lanning and Cohen, 1951) before and after incubation with PAO1 cells in the presence or absence of sanguinarine. As negative control, the test was performed with the Δ*kguT* strain, which is unable to import 2-KG. As shown in Figure 6, the 2-KG concentration in the medium decreased upon incubation with PAO1 cells, whereas it did not change upon incubation with Δ*kguT* cells, as expected. The concentration of the 2-KG remaining in the medium upon incubation with PAO1 was only slightly higher (1.6 ± 0.6-fold) in the presence of sanguinarine than in its absence, suggesting that the 2-KG was transferred from the medium into PAO1 cells also in the presence of the alkaloid. This result contradicts the hypothesis that sanguinarine prevents 2-KG transport by KguT and suggests that sanguinarine may inhibit an intracellular factor specifically involved in 2-KG catabolism.

### Sanguinarine Inhibits Growth on 2-Ketogluconate of *Pseudomonas aeruginosa* Environmental and Clinical Strains

**FIGURE 7:**
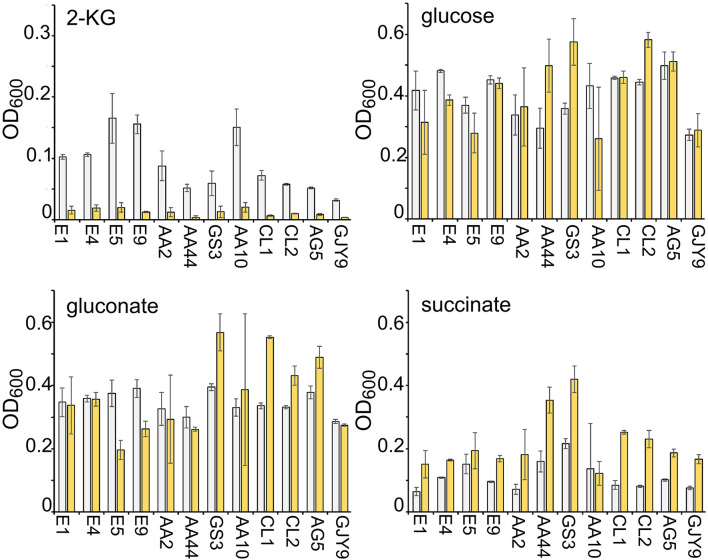
Effect of sanguinarine on the growth of environmental and clinical *Pseudomonas aeruginosa* strains. Growth of the *P. aeruginosa* strains indicated below the bars in M9tx with glucose, gluconate, 2-ketogluconate (2-KG), and succinate, as indicated, without (gray bars) or with (yellow bars) 66.6 μg/ml of sanguinarine. The OD_600_ was measured after 48 h incubation with gentle agitation at 37°C in glass tubes. Bars represent average with standard deviation (*N* = 3).

Five environmental and 15 clinical isolates, mainly derived from pulmonary infections of CF patients, were tested for growth in minimal medium with 2-KG as the unique carbon source in the presence or absence of sanguinarine. An environmental and seven clinical strains were unable to grow on 2-KG (data not shown) and were excluded from further analysis. Sanguinarine inhibited growth of all the other strains on 2-KG, but not on the other tested carbon sources, namely, succinate, glucose, and gluconate (Figure 7). In fact, the growth on succinate of almost all strains and on glucose and gluconate of three and four clinical isolates, respectively, was slightly stimulated in the presence of sanguinarine. Thus, sanguinarine inhibition of 2-KG utilization is not limited to PAO1 strain.

## Discussion

In this work, we identified sanguinarine as a specific inhibitor of 2-KG utilization in *P. aeruginosa*.

Sanguinarine is produced by *S. canadensis* and other plants used in herbal medicine. The rhizome of *S. canadensis* contains many biologically active alkaloids besides sanguinarine, which were studied for their anticancer, antimicrobial, and anti-inflammatory properties (reviewed by Croaker et al., 2016). In particular, the antibacterial activity of sanguinarine against Gram-positive and Gram-negative bacteria has long been known (Dzink and Socransky, 1985). Interestingly, it was demonstrated that sanguinarine induces *Bacillus subtilis* and *Escherichia coli* filamentation by inhibiting the Z-ring formation, an effect that was ascribed by different authors to either direct FtsZ binding by sanguinarine or indirect membrane perturbation (Beuria et al., 2005; Foss et al., 2013). On the other hand, to the best of our knowledge, sanguinarine activity against *P. aeruginosa* had not been reported so far. Our data rule out that sanguinarine, at the concentrations used in this work, may be generically toxic for this bacterium, for instance, through membrane or DNA damage, as suggested for other bacteria or eukaryotes (Croaker et al., 2016; Singh and Sharma, 2018; Zhang et al., 2020; Fu et al., 2021). Indeed, although PAO1 growth in minimal medium with 2-KG as the sole carbon source is inhibited by sanguinarine, the growth of the same strain with glucose is slightly stimulated by sanguinarine, and this effect was observed also with other *P. aeruginosa* strains and other carbon sources like gluconate and succinate (Figures 3A,7).

We applied a two-step protocol to identify specific inhibitor of 2-KG utilization, based on a primary screening to identify molecules inhibiting either the synthesis of 2-KG from gluconate or its uptake and conversion into 6-phosphogluconate, followed by a secondary assay to check the specificity of candidate inhibitors for the 2-KG utilization pathway. In the primary screening, we analyzed the effect of the Prestwick Chemical Library compounds on the growth of the PAO1 Δ*gntP* derivative on gluconate. Such strain cannot import gluconate and must convert it into 2-KG to grow in a medium in which gluconate is the only carbon source. In principle, inhibitors of 2-KG utilization could also have been identified by directly testing in the primary screening the effect of candidate molecules on the growth of PAO1 on 2-KG. However, since Gad is dispensable for PAO1 growth on 2-KG, inhibitors of Gad would have escaped this assay, thus limiting the number of possible targets of the 2-KG utilization branch. Moreover, the protocol that we used was advantageous from a technical point of view, because we observed in preliminary experiments that *P. aeruginosa* growth on 2-KG in the 96-well plate format used for the screening was quite poor with respect to the growth with gluconate (see also Figure 4), thus making less straightforward the detection of the inhibitory effect of sanguinarine on growth.

Sanguinarine inhibited by more than the 80% the growth of the Δ*gntP* mutant on gluconate, whereas it did not affect PAO1 growth in the same medium, making very unlikely the possibility that this alkaloid may have unspecific toxicity toward *P. aeruginosa* at the tested concentration. Sanguinarine also inhibited by about the 40% the Δ*gntP* growth with glucose, at least in 96-well plates. It is tempting to speculate that such inhibition may be due to the Gad- and Gcd-dependent sequential oxidation of glucose into gluconate, which cannot enter the Δ*gntP* cells, and 2-KG, which cannot be catabolized in the presence of sanguinarine. In other words, the activity of Gad and Gcd may lower glucose concentration in the medium by converting (part of) it into two molecules that bacteria cannot use, thus explaining the growth reduction.

We do not have a conclusive evidence about the identity of the actual sanguinarine molecular target in *P. aeruginosa*. Our data rule out the periplasmic Gad protein and the 2-KG transporter KguT, pointing to either KguD or KguK as a possible target. Inhibitors targeting the 2-KG branch of glucose catabolism are particularly interesting because *P. aeruginosa* mutants lacking the KguT transporter are attenuated in *Galleria mellonella*, thus suggesting a possible correlation between 2-KG catabolism and virulence (Raneri et al., 2018). Indeed, a connection between the oxidative pathway of glucose catabolism and the expression of virulence functions like the type III secretion system and exotoxin A has been shown (Colmer and Hamood, 1998; Daddaoua et al., 2012, 2014; O’Callaghan et al., 2012). Unfortunately, low solubility of sanguinarine in water and DMSO toxicity to the larvae (Allegra et al., 2018; data not shown) made it impossible to assess the sanguinarine effect in *G. mellonella* at the concentrations that have *in vitro* anti-*Pseudomonas* activity.

Sanguinarine has a cytotoxic effect on human cells due to its DNA damaging activity and its interference with the function of different RNA and protein targets (recently reviewed by Croaker et al., 2016; Singh and Sharma, 2018). Its toxicity, together with its low water solubility, makes sanguinarine an unsuitable, or at least difficult, candidate for drug development. However, this work demonstrates that it is possible to isolate inhibitors of specific branches of glucose utilization in a pathogen difficult to treat such as *P. aeruginosa* by screening a relatively small collection of compounds with a very simple and economical procedure. It seems sound that the application of the here described screening protocol to chemical collections larger than the Prestwick Chemical Library could result in the discovery of molecules specifically targeting all branches of *Pseudomonas* glucose import and/or catabolism, which could be used in combination therapy with antibiotics. This would be very relevant considering the role played by *P. aeruginosa* in potentially life-threatening infections typical for diabetic patients (Peleg et al., 2007; Treviño González et al., 2021), and its high resistance toward antibiotics.

A previous research of inhibitors of *P. aeruginosa* planktonic growth in complex medium among the compounds of the Prestwick Chemical Library failed, as the only “hits” found were already known antimicrobials of various classes (Torres et al., 2018). Conversely, we found that 30 Prestwick Chemical Library compounds, heterogeneous in both their chemical structures and known pharmacological activities, reduced by at least the 50% PAO1 growth in minimal medium with gluconate and the Δ*gntP* strain growth with glucose or gluconate. In such conditions, the two strains rely on different branches of glucose metabolism, suggesting that the inhibitors may target either enzymes of the Entner–Doudoroff pathway, which are required for the growth on both carbon sources, or biosynthetic pathways dispensable for the growth in complex medium, but essential in minimal medium. Given the central role played by biosynthetic genes in the infection of *P. aeruginosa* and other bacteria (Brown and Stocker, 1987; Crawford et al., 1996; Handfield et al., 2000; Pechous et al., 2006; Dubern et al., 2015), these compounds may deserve further investigations, as they could potentially have an interesting anti-*Pseudomonas in vivo* activity.

## Data Availability Statement

The original contributions presented in the study are included in the article/Supplementary Material, further inquiries can be directed to the corresponding author/s.

## Author Contributions

FB conceived, designed the study, and wrote the manuscript. FAF, GB, GP, MR, MS, and FF performed the experiments. FAF, FF, AC, and FB analyzed the data. All authors critically revised and approved the final version of the manuscript.

## Conflict of Interest

The authors declare that the research was conducted in the absence of any commercial or financial relationships that could be construed as a potential conflict of interest.

## Publisher’s Note

All claims expressed in this article are solely those of the authors and do not necessarily represent those of their affiliated organizations, or those of the publisher, the editors and the reviewers. Any product that may be evaluated in this article, or claim that may be made by its manufacturer, is not guaranteed or endorsed by the publisher.
