# Supplementary material for: Sanguinarine Inhibits the 2-Ketogluconate Pathway of Glucose Utilization in Pseudomonas aeruginosa
Source: Front Microbiol. 2021 Sep 10;12:744458. doi: 10.3389/fmicb.2021.744458 (PMC8461315; doi:10.3389/fmicb.2021.744458)
Supplement: Supplementary file 1 [file Data_Sheet_1.pdf]

*Supplementary material*

**Supplementary Table 1. Compounds passing the primary screening.**

| Name | Growth[a] |
|---|---|
| (+,-)-Synephrine | 0.053 |
| (d,l)- Tetrahydroberberine | 0.051 |
| (R)-Propranolol hydrochloride | 0.067 |
| 3-alpha-Hydroxy-5-beta-androstan-17-one | 0.076 |
| 8-Azaguanine | 0.002 |
| Acacetin | 0.031 |
| Amethopterin (R,S) | 0.053 |
| Amiprilose hydrochloride | 0.077 |
| Amoxapine | 0.025 |
| Astemizole | 0.055 |
| Atovaquone | 0.029 |
| Azacyclonol | 0.000 |
| Benzamil hydrochloride | 0.064 |
| Benzbromarone | 0.040 |
| Bepridil hydrochloride | 0.036 |
| Bromperidol | 0.047 |
| Chlorprothixene hydrochloride | 0.045 |
| Ciclopirox ethanolamine | 0.007 |
| Clemastine fumarate | 0.076 |
| Clioquinol | 0.025 |
| Clomipramine hydrochloride | 0.054 |

| | |
|---|---|
| Colchicine | 0.078 |
| Condelphine | 0.080 |
| Cyclobenzaprine hydrochloride | 0.051 |
| Cyproheptadine hydrochloride | 0.000 |
| Dacarbazine | 0.055 |
| Danazol | 0.082 |
| Dehydrocholic acid | 0.016 |
| Desipramine hydrochloride | 0.056 |
| Dicyclomine hydrochloride | 0.057 |
| Dubinidine | 0.075 |
| Edrophonium chloride | 0.012 |
| Epitiostanol | 0.000 |
| Estrone | 0.046 |
| Etanidazole | 0.049 |
| Fendiline hydrochloride | 0.055 |
| Flucytosine | 0.008 |
| Fluvoxamine maleate | 0.030 |
| GBR 12909 dihydrochloride | 0.080 |
| Gossypol | 0.000 |
| Guanabenz acetate | 0.069 |
| Guanadrel sulfate | 0.070 |
| Hexestrol | 0.018 |
| Homochlorcyclizine dihydrochloride | 0.039 |
| Hycanthone | 0.054 |
| Hydroxytacrine maleate (R,S) | 0.080 |

| | |
|---|---|
| Hydroxyzine dihydrochloride | 0.048 |
| Idazoxan hydrochloride | 0.065 |
| Ifosfamide | 0.065 |
| Iohexol | 0.073 |
| Leflunomide | 0.041 |
| Leucomisine | 0.077 |
| Lorglumide sodium salt | 0.065 |
| Maprotiline hydrochloride | 0.037 |
| Mefloquine hydrochloride | 0.049 |
| Metergoline | 0.058 |
| Methiothepin maleate | 0.035 |
| Molindone hydrochloride | 0.041 |
| Monobenzone | 0.043 |
| Nandrolone | 0.079 |
| Nilutamide | 0.071 |
| Nimodipine | 0.067 |
| Nitrarine dihydrochloride | 0.066 |
| Norcyclobenzaprine | 0.028 |
| Nortriptyline hydrochloride | 0.043 |
| Oxybenzone | 0.061 |
| Paroxetine Hydrochloride | 0.040 |
| Perhexiline maleate | 0.037 |
| Perphenazine | 0.038 |
| Phenazopyridine hydrochloride | 0.058 |
| Phenelzine sulfate | 0.000 |

| | |
|---|---|
| Pimethixene maleate | 0.043 |
| Piroxicam | 0.080 |
| Pizotifen malate | 0.040 |
| Proadifen hydrochloride | 0.047 |
| Procyclidine hydrochloride | 0.052 |
| Proguanil hydrochloride | 0.048 |
| Promazine hydrochloride | 0.047 |
| Promethazine hydrochloride | 0.071 |
| Quinapril HCl | 0.075 |
| Rolipram | 0.022 |
| Sanguinarine | 0.005 |
| Sulconazole nitrate | 0.046 |
| Suxibuzone | 0.063 |
| Terfenadine | 0.020 |
| Triflusal | 0.077 |
| Trigonelline | 0.068 |
| Vidarabine | 0.071 |
| Zuclopenthixol hydrochloride | 0.040 |

[a]Growth of PAMO107 (i.e. PAO1 Δ*gntP*) in M9 supplemented with 0.4% gluconate was estimated as the difference between $OD_{600}$ after 24h incubation at 37 °C and $OD_{600}$ at time 0. The $OD_{600}$ reached by the cultures growing with no compound in this condition was about 0.34.

**Supplementary Table 2. Unspecific growth inhibitors**

| Compound | R[a] | | |
|---|---|---|---|
| | gluconate | | glucose |
| | Δ*gntP* | *gntP*$^+$ | Δ*gntP* |
| Ciclopirox ethanolamine | 1.0 | 1.0 | 1.0 |
| Flucytosine | 1.0 | 0.8 | 0.9 |
| Clioquinol | 0.9 | 1.0 | 0.9 |
| Azacyclonol | 0.9 | 0.9 | 0.7 |
| Azaguanine-8 | 0.9 | 0.6 | 0.9 |
| Cyproheptadine hydrochloride | 0.9 | 0.9 | 0.8 |
| Paroxetine Hydrochloride | 0.9 | 0.9 | 0.9 |
| Atovaquone | 0.9 | 0.5 | 0.7 |
| Bepridil hydrochloride | 0.9 | 0.7 | 0.7 |
| Acacetin | 0.9 | 0.7 | 0.7 |
| Promethazine hydrochloride | 0.8 | 0.8 | 0.8 |
| Fluvoxamine maleate | 0.8 | 0.5 | 0.5 |
| Metergoline | 0.8 | 0.5 | 0.7 |
| Amethopterin (R,S) | 0.8 | 0.5 | 0.6 |
| Promazine hydrochloride | 0.8 | 0.8 | 0.8 |
| Dacarbazine | 0.8 | 0.5 | 0.7 |
| Zuclopenthixol hydrochloride | 0.8 | 0.7 | 0.5 |
| Leflunomide | 0.8 | 0.6 | 0.8 |
| Benzbromarone | 0.8 | 0.6 | 0.6 |
| Hexestrol | 0.8 | 0.5 | 0.9 |
| Proadifen hydrochloride | 0.8 | 0.5 | 0.5 |
| GBR 12909 dihydrochloride | 0.7 | 0.6 | 0.6 |
| Norcyclobenzaprine | 0.7 | 0.8 | 0.8 |
| Edrophonium chloride | 0.7 | 0.6 | 0.7 |
| Monobenzone | 0.7 | 0.6 | 0.7 |
| 3-alpha-Hydroxy-5-beta-androstan-17-one | 0.7 | 0.5 | 0.7 |
| Oxybenzone | 0.6 | 0.5 | 0.7 |
| Phenazopyridine hydrochloride | 0.6 | 0.5 | 0.7 |
| Etanidazole | 0.5 | 0.6 | 0.5 |
| Amiprilose hydrochloride | 0.5 | 0.5 | 0.5 |

[a]R was calculated for each strain/condition with respect to control cultures containing 5 µl of DMSO as explained in "Materials and Methods". R= 1, complete growth inhibition; R = 0, no inhibition. Δ*gntP*, PAMO107; *gntP*$^+$, PAO1.

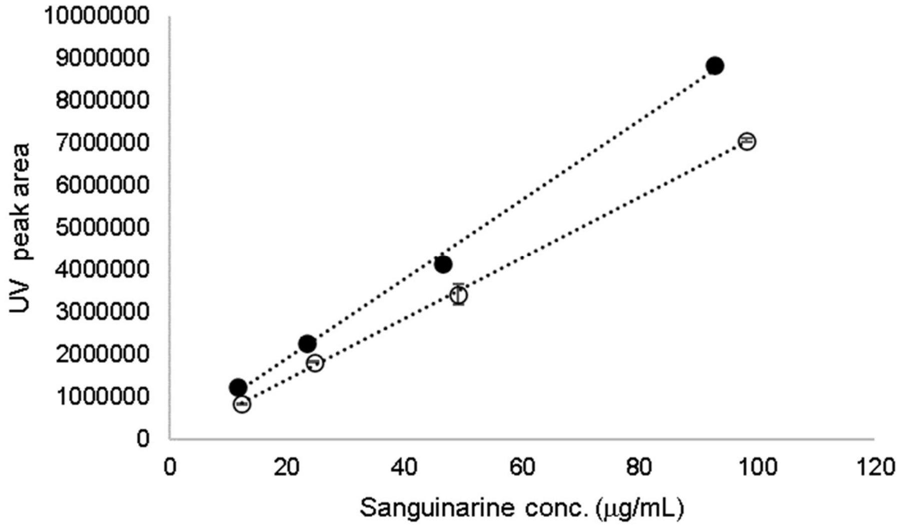


**Supplementary Figure 1.** Calibration curve with cell culture medium used for sanguinarine quantification by HPLC-UV (empty circles, see Materials and Methods), calibration curve in MeOH (solid circles).

A

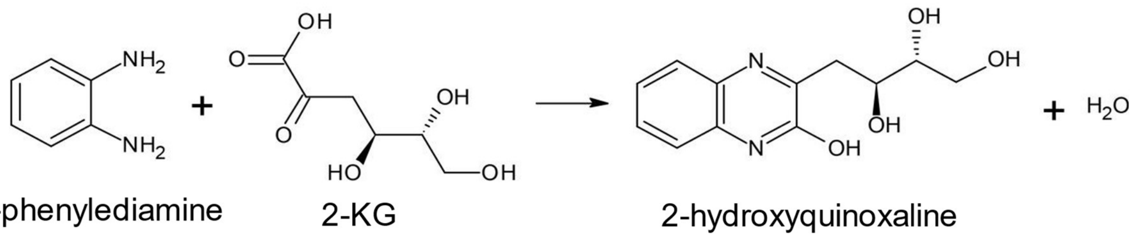


B

| | A330 | | | | | |
|---|---|---|---|---|---|---|
| | + Sanguinarine | | | - Sanguinarine | | |
| 2-KG (µg/ml) | 1 | 2 | M* | 1 | 2 | M* |
| 30 | 5.08 | 5.04 | 2.51 | 2.26 | 2.66 | 2.46 |
| 15 | 3.87 | 3.94 | 1.36 | 1.30 | 1.29 | 1.29 |
| 8 | 3.34 | 3.33 | 0.79 | 0.73 | 0.72 | 0.72 |
| 4 | 2.93 | 2.98 | 0.41 | 0.37 | 0.37 | 0.37 |
| 2 | 2.70 | 2.80 | 0.21 | 0.20 | 0.20 | 0.20 |
| 0 | 2.51 | 2.58 | | 0.00 | | |

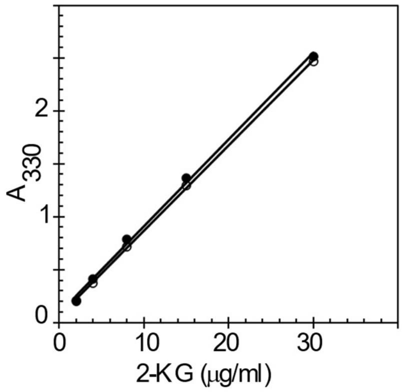


**Supplementary Figure 2. Rationale and setup of 2-KG concentration assay.** A. In the assay conditions (see Materials and Methods), 2-KG reacts with *o*-phenylendiamine generating 2-hydroxyquinoxaline, which absorbs light at 330 nm. B. $A_{330}$ values of samples containing or not sanguinarine and different 2-KG concentrations, as indicated. M*, mean of $A_{330}$ values obtained for the two replicate samples for each 2-KG concentration subtracted with the $A_{330}$ of the samples without 2-KG. In the graph below the table, the means *vs*. 2-KG concentrations were plotted. Empty symbols, samples without sanguinarine; black symbols, samples with sanguinarine.

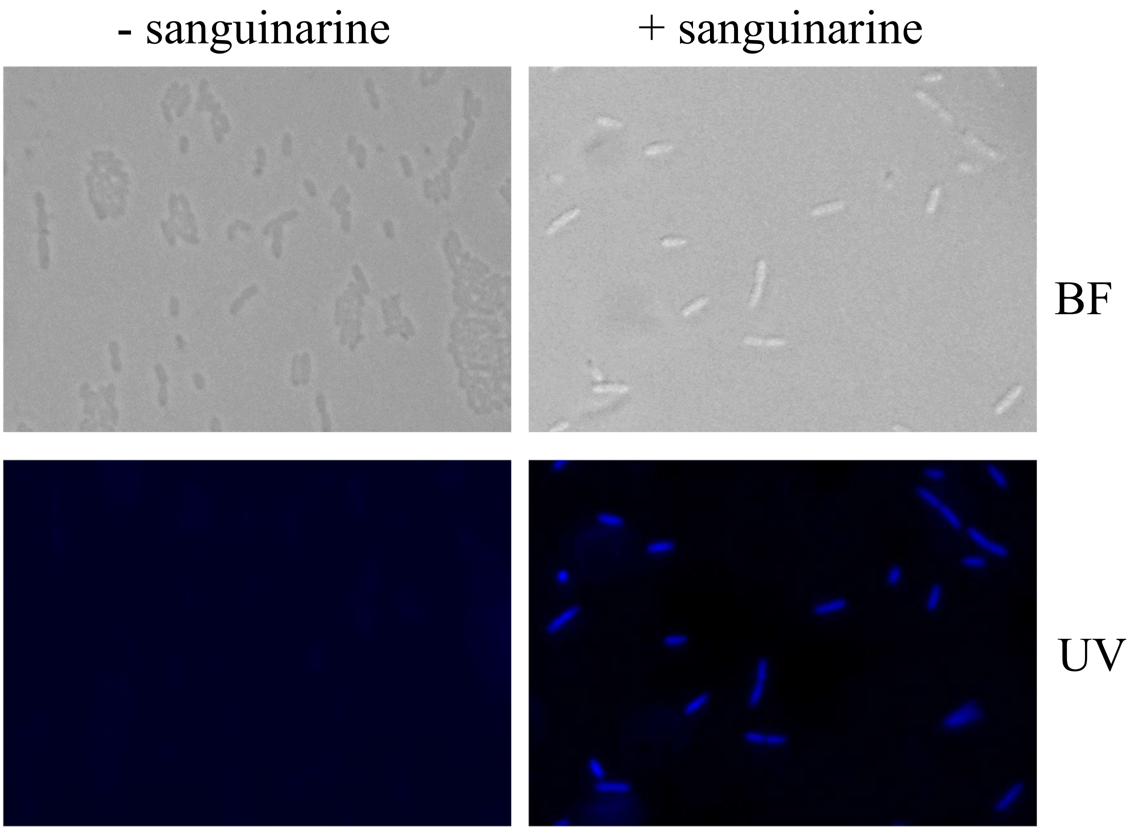


**Supplementary Figure 3. Microscopy observation of PAO1 cells.** Cultures of PAO1 grown in M9tx with 0.4% gluconate with or without 66.6 µg/ml sanguinarine, as indicated, were observed by bright field (BF) or UV microscopy.
